# Mussel byssus-inspired dual-functionalization of zirconia dental implants for improved bone integration

**DOI:** 10.1016/j.mtbio.2024.101007

**Published:** 2024-02-22

**Authors:** Qihong Zhang, Shuyi Wu, Yingyue Sun, Kendrick Hii Ru Yie, Jiatong Zhuang, Tingting Liu, Wen Si, Yinyan Zhang, Zheyuan Liu, Lifeng Xiong, Lei Lu, Peng Gao, Jinsong Liu

**Affiliations:** School and Hospital of Stomatology, Wenzhou Medical University, Wenzhou, 325027, China

**Keywords:** Zirconia implant, Osteogenesis, Cell adhesion, Strontium, Amino groups

## Abstract

Zirconia faces challenges in dental implant applications due to its inherent biological inertness, which compromises osseointegration, a critical factor for the long-term success of implants that rely heavily on specific cell adhesion and enhanced osteogenic activity. Here, we fabricated a dual-functional coating that incorporates strontium ions, aimed at enhancing osteogenic activity, along with an integrin-targeting sequence to improve cell adhesion by mussel byssus-inspired surface chemistry. The results indicated that although the integrin-targeting sequence at the interface solely enhances osteoblast adhesion without directly increasing osteogenic activity, its synergistic interaction with the continuously released strontium ions from the coating, as compared to the release of strontium ions alone, significantly enhances the overall osteogenic effect. More importantly, compared to traditional polydopamine surface chemistry, the coating surface is enriched with amino groups capable of undergoing various chemical reactions and exhibits enhanced stability and aesthetic appeal. Therefore, the synergistic interplay between strontium and the functionally customizable surface offers considerable potential to improve the success of zirconia implantation.

## Graphical abstract

**Figure:**
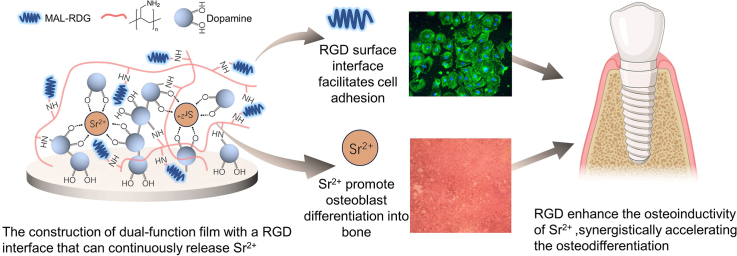

## 1 Introduction

In dental implantology, titanium implants have always been the gold standard due to their excellent biocompatibility and mechanical properties [1]. However, reports of adverse allergic reactions and suboptimal aesthetics with titanium implants underscore the need for a more biologically active and aesthetically superior substitute for dental implant materials [2]. Zirconia, a type of bioceramic, has emerged as a promising alternative dental implant due to its excellent aesthetic, and optical properties [3]. However, it is widely acknowledged that zirconia implants have a significantly higher early failure rate compared to titanium implants, primarily due to their bio-inertness which impedes the adhesion and differentiation of bone cells, crucial for rapid formation of bone tissue and achieving a tight integration with the implant [4,5]. Thus, enhancing the osseointegration capacity of zirconia implants through various surface modifications, employing physical or chemical methods, is crucial and beneficial for their long-term success in implant restoration [6].

Over the past decade, various techniques such as sandblasting, acid etching, magnetron sputtering, plasma deposition, and layer-by-layer self-assembly procedures have been employed for material surface bioengineering [[7], [8], [9]]. However, reported drawbacks include the long-term inefficiency of physical modification procedures and the detrimental impact of chemical surface treatments on the function of bioactive molecules [[10], [11], [12]]. In addition, these methods also suffer from drawbacks such as low controllability and unstable operability [13]. Therefore, there is a crucial need to explore simpler and more feasible strategies for the functionalization of surfaces.

Fortunately, the development of biomimetic technology offers a more viable and advantageous approach to surface bioengineering for the integration of individual components or combinations of these components onto the implant surfaces in a more flexible and convenient manner [[14], [15], [16], [17]]. Inspired by mussel adhesion, Lee et al. reported a novel approach to fabricating a multifunctional polydopamine (PD) coating by dip-coating an object into an aqueous solution of dopamine [18]. The synergistic interaction between catechol and amines allows dopamine to assemble and form PD coating on various material surfaces, including ceramic materials, such as zirconia [19,20].

However, it's important to note that PD coating only biomimics the adhesive properties of mussel foot thread adhesion plaques [21]. The PD coating is formed by the oxidative polymerization of dopamine into various oligomers, primarily stacking through non-covalent bonds, which poses a challenge in terms of the coating's durability and longevity [22]. Secondly, the secondary grafting of functional molecules onto the PD coating surface is limited, requiring the presence of thiol and amine groups [23]. Fortunately, the outer cuticle of mussel byssal threads, needing protection from abrasion in wave-swept habitats, is 5-fold stronger than its core due to the incorporation of iron ion coordination in protein (that is rich in the catecholic amino acid 3,4-dihydroxyphenylalanine) cross-linking. This inspires us to design more suitable surface modifications for implants [[24], [25], [26]].

**Scheme 1:**
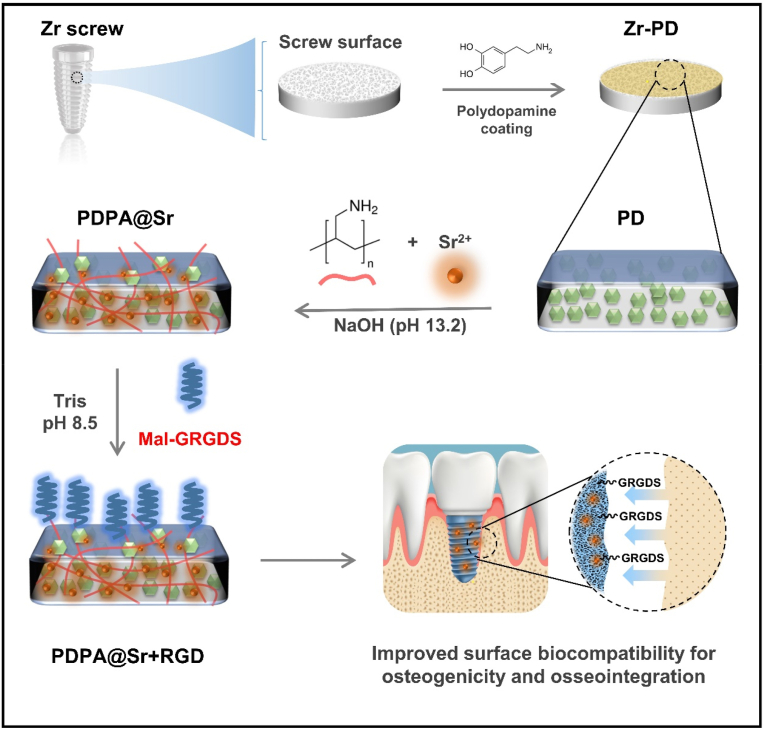
Schematic illustration of modification process on zirconia implant surface.

Here, we employed a strong base to disrupt the non-covalent bonds in the PD coating, allowing it to be modulated by ions and molecules. At the same time, we selected strontium ions and polyallylamine (PAH) which contains numerous ethylamine groups, both stable in alkaline solutions, to regulate the structure of the PD coating. Through a simple one-step immersion, a coating enriched with strontium ions and abundant in surface amine groups can be obtained. The Sr^2+^ ions, having the ability to stimulate osteoblast formation [27], also coordinate with the catechol residues to create an environment to better facilitate osteogenesis. Research has shown that surfaces constructed with grafted RGD peptides can specifically adhere to cells and, in synergy with osteogenic peptide factors, enhance the osseointegration of implants [28]. To further enhance the binding of new bone to the surface of the implant, we introduced polypeptide RGD by taking advantage the abundant amino groups through a mild and biocompatible maleimide-amine addition reaction. Following the successful synthesis of the implant, both *in vitro* and *in vivo* investigations were carried out to explore the synergistic effects of Sr^2+^ as an osteogenic agent and the cell adhesion polypeptide RGD on interface osteogenesis (Scheme 1). More importantly, compared to PD coating, the dual-functional coating surface is rich in amine groups capable of undergoing various chemical reactions, allowing for the grafting of a range of functional molecules including RGD and exhibits more aesthetically pleasing characteristics and improved stability. By studying the performance and outcomes of the engineered implant in these contexts, we sought to demonstrate the positive impact of this design on osseointegration. We anticipate that this approach will offer a direct and highly effective solution for enhancing osseointegration of zirconia implants.

## 2 Materials and methods

### 2.1 Sample preparation

Zirconia bioceramic blocks (ZrO_2_; 3Y-TZP, UPCERA, China) were cut into ZrO_2_ discs (10 mm diameter, 1.5 mm thickness) and cylindrical ZrO_2_ implants (5 mm diameter, 10 mm length). Samples were then sintered at 1500 °C for 2 h. Subsequently, 400, 600, and 1000 mesh sandpapers were used to polish the zirconia substrates, followed by a sequential 15 min ultrasonic cleaning in acetone, ethanol, and deionized water, respectively. In brief, the polished zirconia samples were initially immersed in a dopamine hydrochloride solution (Tris buffer, 1.21 mg/mL, pH 8.5) with a final concentration of 1 mg/mL at room temperature for 48 h to form a polydopamine (PD) coating. Then, the PD-coated ZrO_2_ was immersed in a 1:1 vol ratio of SrCl_2_ (0, 20 mg/mL, Macklin, China) and NaOH (6 mg/mL, containing 2% PAH, Macklin China) solution at room temperature for 12 h to obtain PAH cross-linked PD coating (denoted as PDPA) and PAH and Sr cross-linked PD coating (PDPA@Sr, denoted as Sr) samples. Mal-GRGDS is one of the RGD peptide, and in subsequent experiments, we refer to the RGD peptide as GRDGS peptide. Finally, these samples were soaked at room temperature for 6 h in pH 8.5 Tris buffer solution containing Mal-GRGDS peptide (1 mg/mL, Synthesized by Sci Bio, China) to obtain PDPA-GRGDS coating (denoted as RGD) and PDPA@Sr-GRGDS coating (denoted as Sr + RGD) samples. Each step of the samples was thoroughly washed with ultrasonication to remove loosely bound substances, followed by drying and storage in a vacuum-sealed container.

### 2.2 Characterization of the surface physicochemical properties

The topological cues and characterizations of the samples were assessed and observed using scanning electron microscopy (SEM, HITACHI SU8010, Japan) and atomic force microscopy (AFM, Bruker Dimension ICON, USA). To evaluate the hydrophilicity of the sample surface, a contact angle measuring instrument (DSA30, Kruss, Germany) was employed. The chemical composition of the samples was analyzed using X-ray photoelectron spectroscopy (XPS, Thermo Kalpha, USA) and the energy-dispersive X-ray spectroscopy (EDS, Phenom Pharos, Netherlands).

### 2.3 The determination of color different

Surface color of different samples was captured using an ordinary camera (Canon EOS 800D, Japan). Subsequently, the color difference was calculated relative to the reference color of zirconia.

$$\Delta E=\sqrt{\Delta L^{2}+\Delta a^{2}+\Delta b^{2}}$$

Here, ΔE represents the color difference, ΔL, Δa, and Δb respectively represent the differences between two samples in brightness (L), the red-green axis (a), and the blue-yellow axis (b).

### 2.4 RGD peptide determination

To quantify the amounts of GRGDS peptide on the coating, toluidine blue reagent was used. Electrostatic force can facilitate the bonding of toluidine blue to the carboxyl group in GRGDS, enabling its use for assessing the concentration of GRGDS on the sample's surface. The samples were immersed in a 0.01 mol/mL solution of toluidine blue for 4 h at room temperature, followed by washing with deionized water to remove any excess dye until the solution became clear. After taking photographs, 350 μL of HCl (pH 4) was added, and the solution was shaken for 30 min to dissolve. Then, 300 μL was aspirated into a 96-well plate, and a standard curve was prepared. The OD values were measured at 600 nm by using a microplate reader (Bio-Rad 680, USA).

### 2.5 Release profiles of Sr^2+^

To quantify the release of Sr^2+^, six samples from both the Sr and the Sr + RGD groups were immersed in 5 mL of PBS solution at room temperature. The PBS solution was then collected and replaced with fresh PBS solution at specific time intervals (day 1, 3, 7, 14, and 21). An inductively coupled plasma (ICP, PerkinElmer 8300, USA) was used to analyze and measure the concentration of the Sr^2+^ ions.

### 2.6 Bond strength of coating

The nano-scratch test involved applying a gradually increasing force on the coating using a nano-scratch meter (Bruker Hysitron TI980, Germany) to create a mark, and then determining the bonding force of the coating by analyzing the scratch's shape and depth.

### 2.7 *In vitro* evaluation

#### 2.7.1 Cell culture

The murine pre-osteoblast cell line, MC3T3-E1 (ATCC, China), was cultured in α-MEM medium (Gibco, USA) supplemented with 10% FBS (Gibco, USA), 1% penicillin/streptomycin (PS, Gibco, USA) at 37 °C under 5% CO_2_ environment. Cell passage is performed when the density of MC3T3-E1 cells has reached 80–90% confluence. All samples were sterilized under UV exposure and immersion in 75% ethanol solution before any *in vitro* experiments.

#### 2.7.2 Cell viability and proliferation assay

MC3T3-E1 cells at a density of 2 × 10^4^ cells/cm^2^ were seeded onto the different samples (6 samples/group) in a 24-well plate. After culturing in growth medium at 37 °C and 5% CO_2_ for 1, 4, 7 days, the cell viability and proliferation was evaluated using the Cell Counting Kit 8 (CCK-8, NCM, China). In brief, 300 μL of the CCK-8 solution (α-MEM medium + 10% CCK-8) was added into each well and were incubated at 37 °C for 30 min. Then, 100 μL of the solution was transferred into a 96-well plate, and the absorbance was measured at 450 nm.

#### 2.7.3 Early cell adhesion and cell morphology

The adhesion of MC3T3-E1 cells was assessed through fluorescence staining. Initially, the cells at a density of 2 × 10^4^ cells/cm^2^ were seeded and cultured on the different samples in a fetal bovine serum (FBS)-free α-MEM medium (6 samples/group). After incubation for 3 h, the cells were taken out and washed 3 times using PBS solution. The adherent cells were then fixed with 4% paraformaldehyde solution (Beyotime, China) for 40 min, immersed in a 1% (v/v) Triton X-100 (Beyotime, China) solution for cell membrane perforation. Next, a double-dye staining of FITC-labeled Phalloidin (Solarbio, China) and 4′,6-diamidino-2-phenylindole hydrochloride (DAPI, Solarbio, China) were used to stain the cytoskeletal actin and cell nuclei, respectively. Stained cellular morphology were observed and analyzed under fluorescence microscope (NIKON, Japan). Five visual fields on each sample were randomly selected and viewed. Subsequently, the Image J software was used for analysis.

#### 2.7.4 Alkaline phosphatase (ALP) activity and staining

To investigate the differentiation potential of MC3T3-E1 cells, the ALP activity was determined. Cells were seeded onto the corresponding samples at an initial density of 2 × 10^4^ cells/cm^2^ (6 samples/group). The preparation for ALP staining is done on day 7 post-seeding. Initially, the cells were fixed with 4% paraformaldehyde for 15 min and stained using BCIP/NBT Alkaline Phosphatase Color Development Kit (Beyotime, China). The stained images were viewed under a stereomicroscope (Olympus, Japan).

The quantification of ALP activity was determined after 7 days of culture. To begin, the cells were lysed with the 1% Triton X-100 solution for 30 min. Next, the BCA protein assay kit (NCM, China) as well the ALP assay kit (Beyotime, China) was prepared to determine total protein concentration and ALP activity. Their absorbance value was measured using a microplate reader at 562 nm and 520 nm, respectively. The final ALP activity was normalized with the total protein concentration.

#### 2.7.5 Mineralization

MC3T3-E1 cells were cultured onto the various samples at a density of 2 × 10^4^ cells/cm^2^ for 14 days (6 samples/group). Afterwards, the cells were initially immobilized with 4% paraformaldehyde for 30 min followed by alizarin red S staining (ARS, Solarbio, China) for another 30 min. ARS was employed to stain the calcium nodules on the substrate. A stereomicroscope was used for the observation and viewing of the stained samples. In order to quantify the mineralization activity, the samples were immersed into 10% cetylpyridinium chloride solution for 30 min to dissolve the calcium nodules. The solution was then transferred into a 96-well plate and the absorbance value was measured using a microplate reader at 520 nm.

#### 2.7.6 Expression of osteogenic genes

MC3T3-E1 cells (2 × 10^4^ cells/cm^2^) were cultured onto the different samples (4 samples/group). At day 7, the cells were lysed using Trizol and the total RNA was extracted using an RNA simple Total RNA Kit (TIANGEN, China) according to manufacturer's protocol. Subsequently, the collected RNA was reverse transcribed into cDNA by using a PrimeScript RT reagent Kit (TAKARA, Japan). To detect the expression of osteogenesis-related genes, the qRT-PCR was performed by using SYBR Premix ExTM *Taq*II (TAKARA, Japan) with specific primers. The primer sequences for the target genes are listed in Table 1.

**Table 1:** Primers of target and housekeeping genes.

Targets	Primers
ALP	F:5′- GAACAGAACTGATGTGGAATACGAA -3′
	R:5′- CAGTGCGGTTCCAGACATAGTG -3′
OCN	F:5′- GAACAGACAAGTCCCACACAGC -3′
	R:5′- TCAGCAGAGTGAGCAGAAAGAT -3′
OPG	F:5′- GCCCAGACGAGATTGAGAG -3′
	R:5′- CAGACTGTGGGTGACGGTT -3′
Runx2	F:5′- AGAGTCAGATTACAGATCCCAGG -3′
	R:5′- TGGCTCTTCTTACTGAGAGAGG -3′
GAPDH	F:5′- CTCGTCCCGTAGACAAAATGGT -3′
	R:5′- GAGGTCAATGAAGGGGTCGTT -3′

### 2.8 *In vivo* evaluations

#### 2.8.1 Animal models

The *in vivo* study was conducted in accordance to the rules set by the Animal Ethics Committee of Wenzhou Institute UCAS (No. WIUCAS22111501). Fifteen female New Zealand white rabbits (6 months old, ±3 kg) were provided by the Wenzhou Institute UCAS for the experiment. The rabbits were randomly allocated into the 3 groups: (1) ZrO_2_; 2) Sr; 3) Sr + RGD. All rabbits were subjected to general anesthesia using prior to surgical procedures. A bilateral lateral longitudinal incision of 15 mm was made on the outer side of the patellar ligament of both hind legs of all rabbits, exposing the lateral femoral condyle. Subsequently, a cylindrical bone defect measuring 4 mm in diameter and 8 mm in depth was created using a 4 mm drill bit. The fragmented bone pieces were thoroughly irrigated with saline solution before the placement of the implants. The surgical site was then closed using sutures and penicillin was administered for the following 3 days post-surgery to prevent infection. The rabbits were humanely euthanized 8 weeks post-implantation and their hind femurs were harvested for histological staining and implant pull-out testing.

#### 2.8.2 Histological analysis

In brief, the bones were initially preserved and fixed by 4% paraformaldehyde, and then cleaned, dehydrated and permeated before being embedded. The sample was cut along the long axis using the hard tissue microtome (EXART CP300) to obtain a sample slice (200 μm thickness). Subsequently, the prepared tissue sections were stained with hematoxylin and eosin (HE) and Masson's trichrome stains and viewed under fluorescence microscope for histological observations.

#### 2.8.3 Implant pull-out testing

To further investigate the mechanical stability of different implants, the biomechanical pull-out tests were conducted. Implants were pulled out with a displacement rate of 10 mm/min using an electronic universal testing machine (MTS EXCEED MODEL E43, China). The test was stopped when the implant was completely separated from the bone, and the maximum load force was recorded.

### 2.9 Statistical analysis

All experiments in this study were conducted with a minimum of 3 repetitions. The experimental data recorded as the mean ± standard deviation (SD). If the data followed a normal distribution, statistical analysis was given by the one-way analysis of variation, considering values of p < 0.05 a significant difference.

## 3 Results

### 3.1 Characterization of the dual-functionalization coating

The Mal-GRGDS (Maleimide-Glycine-Arginine-Glycine-Aspartic Acid-Serine) peptides were prepared by standard Fmoc-based solid-phase peptide synthesis strategy [29]. After purification, high-performance liquid chromatography (HPLC) revealed a purity of 98% (Fig. S1), with further characterization conducted using electrospray ionization mass spectrometry (ESI-MS). The monoisotopic mass [M − H]^−^ of Mal-GRGDS were measured at 642.31, which were calculated to be 641.70 (Fig. S2).

Previous studies have indicated that although PD can firmly adhere to material surfaces through a simple method, it often results in a discernible alteration in the color of ZrO_2_, leading to a darker appearance [30]. As shown in Fig. S4A, both the PDPA group and the RGD group exhibit colors almost similar to ZrO_2_, while the color change of the Sr group and the Sr + RGD group is considerably less compared to PD. Moreover, this observation is further supported by a quantitative assessment of color differences (Fig. S4B).

**Fig. 1:**
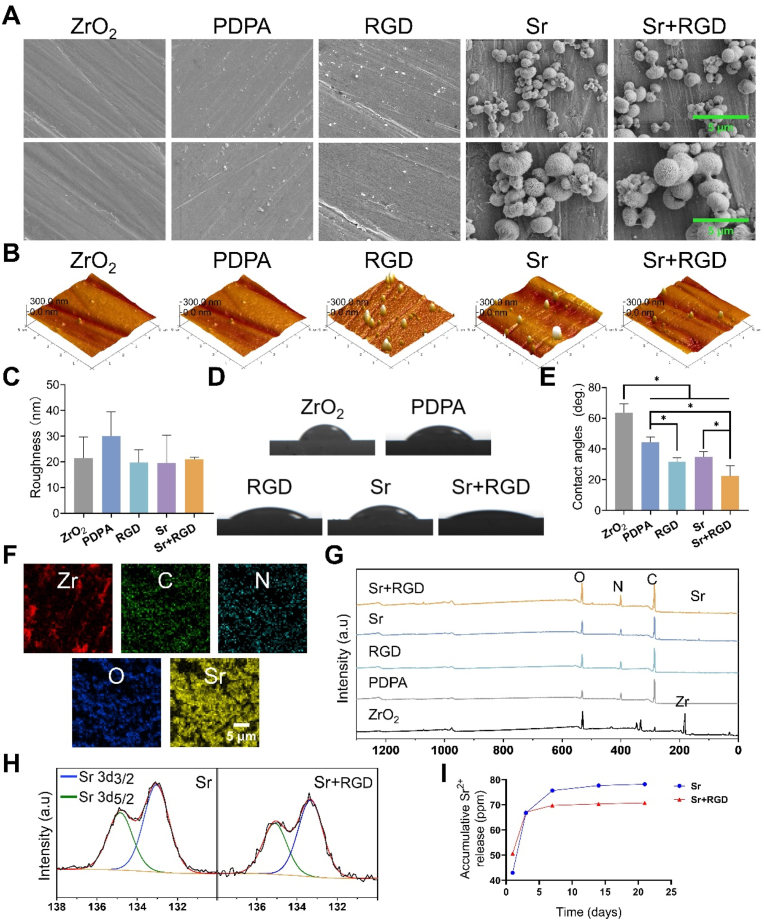
(A) Representative SEM images of different samples. (B) AFM images of different samples and (C) quantitative statistics of surface roughness. (D) The water contact angles of different surfaces and (E) the quantitative results. (F) SEM-EDS elemental mapping for the Sr and Sr + RGD group. (G) XPS full spectra and (H) Sr high-resolution spectrum of different samples. (I) Release profiles of Sr^2+^ from Sr and Sr + RGD substrates at different durations (1, 3, 7, 14 and 21 days).

As depicted from the SEM images (Fig. 1A), the surface of the ZrO_2_ appeared smooth at both low and high magnifications. The scratches were primarily attributed to mechanical polishing. After the modification of PDPA and grafting of GRGDS peptides, it can be observed that there are some aggregated nanoparticles, which are nearly identical to those seen in polydopamine coatings [31]. However, numerous micro/nano-spheres were observed on the surface of the substrates after the doping of Sr^2+^. The surface topography and hydrophilic nature plays a pivotal role in modulating cellular behavior, as enhanced hydrophilicity facilitates protein adsorption and cell adhesion, thereby expediting osseointegration [32]. To further investigate the differences in surface roughness and wettability among the samples, AFM and WCA analyses were carried out and the quantified analyses are presented in Fig. 1B–E. The results indicated that there was no statistical difference in surface roughness after surface modifications (Fig. 1C). However, the modified zirconium groups, especially the Sr^2+^ and GRGDS-modified groups, showed a significantly improved surface wettability due to the hydrophilic effect of surface GRGDS peptides (Fig. 1D and E). The GRGDS peptide chosen in this study contains two carboxyl groups, leading to a significant reduction in water contact angle after surface grafting of GRGDS. Based on the principle that toluidine blue can electrostatically bind to carboxyl groups under weak acidic conditions, and this interaction is disrupted in weak alkaline environments, we performed a quantitative analysis of the GRGDS peptide. As shown in Fig. S5, the results indicate that RGD and Sr + RGD have been successfully grafted onto the PDPA and PDPA@Sr coating surfaces, with grafting densities of 10.84 ± 3.11 and 19.18 ± 2.66 nmol/cm^2^, respectively. The results from EDS (Energy-Dispersive X-ray Spectroscopy) elemental mapping indicate a high concentration of strontium elements within the micro and nano particles on the surface. (Fig. 1F). Furthermore, XPS analysis was carried out to further determine the compound constituent in all of the samples (Fig. 1G). The surfaces of both the Sr group and the Sr + RGD group were characterized by the corresponding Sr 3d_3/2_ and Sr 3d_5/2_, peaks located at ∼135 and 133 eV, respectively (Fig. 1H) [33]. Next, XPS chemical composition statistics (Table S1) displayed that the oxygen content in the RGD and Sr + RGD groups showed an increase of 5.17% and 2.82%, respectively, when compared to the PDPA and Sr groups. This may be attributed to the successful grafting of GRGDS as evidenced by a post-H removal atomic ratio of 26.7% for O element in Mal-GRGDS. As for the Sr^2+^ release, we were able to observe a rapid release of the ion in the first 3 days followed by an abrupt reduction yet gradual release rate in both the Sr and Sr + RGD groups (Fig. 1I). However, the total release rate of Sr^2+^ was significantly higher in the Sr group when compared to the Sr + RGD group, which was recorded at 78 ppm and 70 ppm, respectively.

**Fig. 2:**
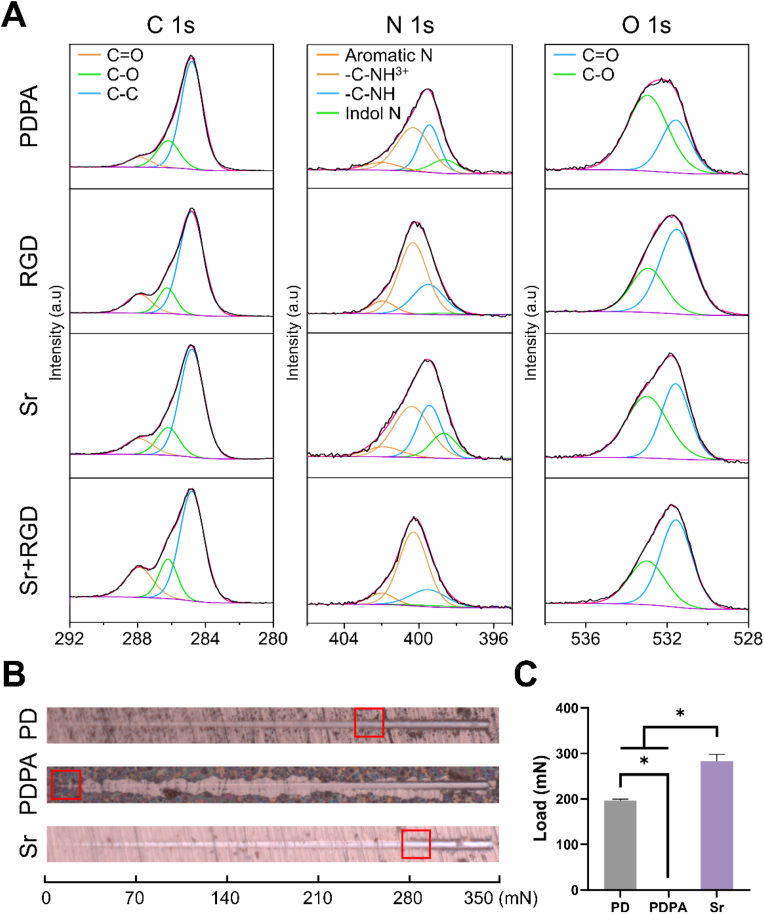
(A) High-resolution XPS spectra of C 1s, N 1s, and O 1s regions for the various samples. (B) SEM representations depicting the morphologies of scratches on various coated surfaces, (C) accompanied by the corresponding load values obtained from the scratch tests.

To further investigate the role of PAH and Sr^2+^ in the coating and the mechanism behind surface-immobilized GRGDS, the high-resolution spectrum of C 1s, O 1s, N 1s were further curve fitted (Fig. 2A). The peak positions (listed values ± 0.2 eV) and atomic concentrations of the functional groups were listed in Table 2. The C 1s spectra were fitted with three components assigned to C–C at 284.8 eV, C–O at ∼286.4 eV, C=O at ∼288.0 eV [34]. The O 1s spectra were fitted with two major peaks at 531.5 and 533.0 eV. The peak at 531.5 eV is assigned to oxygen in C=O, and its proportion increases following GRGDS grafting, which can be attributed to the carbonyl oxygen in GRGDS. The N 1s spectra could be divided into four peaks matching indole N, aromatic N, C–NH, C–NH_3_^+^, centered at ∼398.6 eV, ∼399.5 eV, ∼400.3 eV, ∼402.0 eV, respectively. In the PD coating, the ratio of C–O bonds is significantly higher than that of C=O bonds [31,35] due to the presence of a large number of non-covalent bonds such as hydrogen bonds. However, in the PDPA coating, the proportion of C–O bonds decreases while that of C=O bonds increases. This change is likely due to the disruption of non-covalent bonds in PD coating by OH^−1^, and the crosslinking of dopamine and its oligomers through covalent bonds by PAH. In the oxygen-rich, strongly alkaline solution, more catechol is oxidized to quinone. In addition, it should be noted that compared to PD, the proportion of aromatic –N in PDPA increases, suggesting that the reaction of surface PAH with dopamine and its oligomers is likely a Michael addition. The results of the Sr 3d peak position and the main splitting peaks, Sr 3d₅/₂ and Sr 3d₃/₂, indicate that the PDPA@Sr coating contains Sr^2+^ (Fig. 1H). Despite the coordination interaction between Sr^2+^ and catechol groups being stronger than cation-π interactions, if only coordination were present, the Sr^2+^ would further inhibit the oxidation of catechol into quinone. However, when compared to the PDPA coating, the catechol groups in the PDPA@Sr coating undergo more oxidation, resulting in a decrease in the C–O ratio and an increase in the C=O ratio (Fig. 2 O1s). This may be attributed to the much higher molar concentration of Sr^2+^ in the reaction solution compared to the concentration of catechol. In addition to coordination interactions, the incorporation of Sr^2+^ into the coating may involve cation-π interactions, making it easier for the coating to release strontium ions (Fig. 1I).

**Table 2:** XPS atomic concentration of functional groups in all atoms.

	Peak positions, eV	ZrO_2_	PDPA	RGD	Sr	Sr + RGD
**C 1s**	284.8 (C–C)	0.24	0.56	0.52	0.48	0.42
	286.4 (C–OH)	0.02	0.14	0.1	0.11	0.11
	288.0 (C=O)	0	0.05	0.1	0.08	0.13
**N 1s**	398.6 (indol N)	0	0.02	0	0.02	0
	399.5 (aromatic N)	0	0.04	0.04	0.04	0.03
	400.3 (C–NH)	0	0.06	0.08	0.06	0.09
	402.0 (C–NH_3_^+^)	0	0.01	0.01	0.01	0.01
**O 1s**	532.1 (ZrO_2_)	0.18	0	0	0	0
	531.5 (C=O)	0	0.04	0.11	0.09	0.13
	533.0 (C–O)	0.18	0.07	0.05	0.06	0.05

The atomic concentration of each functional group = the percentage of each functional group in corresponding element × the atomic concentration of the corresponding element in all atoms.

The durability of coatings is paramount for the successful integration of implants. To assess the adhesion of the coatings, we conducted nano-scratch test, where the initial peeling force required to remove the coating is indicated by the red frames on the scratch tracks (Fig. 2B and C). Surprisingly, the PDPA group appears to have minimal binding with ZrO_2_. This phenomenon is improved in the Sr group, with an adhesion force of 283.70 ± 14.59 mN, exceeding that of PD (196.70 ± 2.70 mN). The phenomenon can be attributed to the rapid dissociation of dopamine following the addition of NaOH during the sample preparation process. This rapid dissociation leads to the formation of a thin coating due to limitations in molecular reassembly. On the other hand, Sr^2+^ play a pivotal role by neutralizing NaOH, thus achieving a balance between dissociation and reassembly, and facilitating the coordination of a greater number of strontium ions.

### 3.2 *In vitro* evaluation of osteoblast adhesion and proliferation

**Fig. 3:**
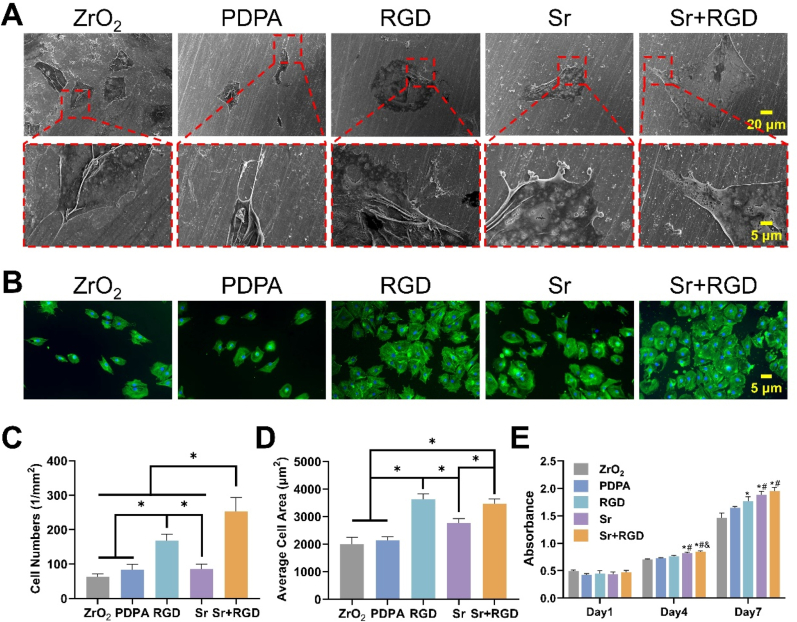
(A) SEM images of the MC3T3-E1 cells on different surfaces for 3 days. (B)The morphologies of early adherent MC3T3-E1 after 3 h of culture on different surfaces (Blue, DAPI; Green, FITC-conjugated phalloidin). (C) and (D) Quantification of adhered cells number and spreading area. (E) The proliferation of MC3T3-E1 cells (Compared with ZrO_2_, the data are mean ± SD, *p < 0.05; Compared with PDPA #p < 0.05; Compared with RGD, &p < 0.05). (For interpretation of the references to color in this figure legend, the reader is referred to the Web version of this article.)

Mouse-derived osteoblast pre-cursor cells, MC3T3-E1, were used to investigate the biocompatibility of the modified samples. Firstly, SEM was employed to analyze the morphology of the substrate-adhered cells. Through the SEM images (Fig. 3A), we noticed that the cells present on the ZrO_2_ and PDPA groups, exhibit a more elongated morphology and a less obvious protrusion of filopodia when compared to the cells on the RGD, Sr, and Sr + RGD substrates. This suggests that the latter three groups facilitated for a better cell spreading. Further investigation using phalloidin-FITC/DAPI staining (Fig. 3B) also confirmed that the cells on the ZrO_2_ surface had a relatively spherical morphology with almost no filopodia extrusion at 3 h of culture while the cells on the RGD, Sr, and Sr + RGD groups exhibited a more polygonal morphology and showed better filopodia extrusion as well as cell adhesion. The observations mentioned above were quantified and tabulated in Fig. 3C and D, we were able to determine that the number of adhered cells as well as the cell spreading were the best in the RGD and the Sr + RGD groups.

Subsequently, CCK-8 assay was used to assess the proliferation and viability of the MC3T3-E1 cells. As shown in Fig. 3E, no obvious cytotoxicity can be seen in all of the groups. We were also able to observe that all groups followed an increasing trend in terms of cellular proliferation potential. Having said that, however, the CCK-8 results for the RGD, Sr, and Sr + RGD groups were significantly higher than that of the ZrO_2_ group at day 7. It should be noted that even though all modified substrate showed better effects on cell proliferation, an intergroup comparison between the RGD, Sr, and Sr + RGD groups suggests that Sr^2+^ was more conducive to MC3T3-E1 proliferation whereas RGD improves cell spreading and adhesion.

The adhesion of osteoblasts onto the surface of implants occurs before their proliferation and differentiation and is a key step in bone tissue formation. GRGDS contains reiterated arginine-glycine-aspartic acid sequences that serves as a specific ligand for integrins on cellular membranes to facilitate cellular adhesion onto the substrates and further promote their differentiation [36]. This interaction facilitates cell adhesion to the substrate and subsequent cytoskeletal rearrangement, thereby augmenting the expression of osteoblast-specific integrins and ultimately promoting osteoblast differentiation [37]. Nevertheless, further investigations are warranted to elucidate the precise underlying mechanism. Conversely, Sr^2+^ demonstrates greater advantages in promoting cell proliferation [38]. With that said, biomimetic peptides and Sr^2+^ modified substrates could effectively improve the cytocompatibility of zirconium, and synergistically promote cell adhesion, spreading and proliferation.

### 3.3 Osteogenic properties *in vitro*

**Fig. 4:**
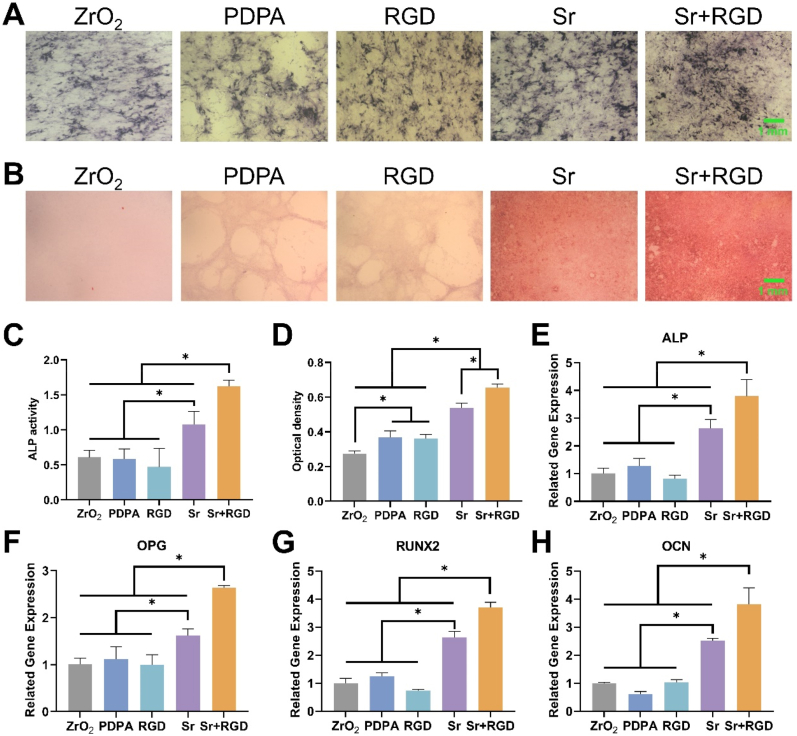
(A) Representative images of ALP staining of MC3T3-E1 cultured on different surfaces after 7 days of culture (B) Representative images of ARS staining after 14 days of culture. (C) Quantitative analysis of ALP activity. (D) Quantitative analysis of ARS staining. (E–H) Relative mRNA expression of ALP, OPG, RUNX2, OCN.

Osteogenic differentiation was first characterized by the activity of ALP, an early marker for assessing the metabolic activity of osteoblasts. In the ALP staining images (Fig. 4A), we can observe that all groups showed a similar degree of staining signifying that all groups were able to facilitate osteogenesis. However, the quantified ALP activity results depicted in Fig. 4C determined that the Sr^2+^-grafted groups (Sr and Sr + RGD groups) were significantly higher when compared to the non-Sr^2+^-grafted groups, with Sr + RGD group being the most conducive to osteogenesis. Likewise, a similar trend could also be observed at the later stages of differentiation. After 14 days of culture, the stained matrix (Fig. 4B) of the Sr + RGD group showed the most and largest mineralized calcium nodes followed by the Sr group. In addition, the quantitative analysis also further confirmed the mineralization capacity of Sr + RGD was 2.3-fold and 1.2-fold higher than that of ZrO_2_ group and Sr group, respectively (Fig. 4D). Apart from the above experiments, we also studied the expression levels of osteogenesis-related genes, including ALP, OPG, RUNX, and OCN (Fig. 4E–H). As expected, the mRNA expression of these osteogenesis-related genes was significantly higher in the Sr + RGD group than the other groups, confirming its remarkable ability to enhance osteogenic differentiation. The gene expression results were consistent with the ALP and mineralization results, and the combination of Sr and GRGDS could synergistically enhance osteogenic differentiation (Fig. 4E). It has been reported that Sr^2+^ significantly promotes osteogenic properties through a calcium-sensing receptor (CaSR) -dependent mechanism, which is attributed to the structural similarity between Sr^2+^ and Ca^2+^ [39,40]. Nonetheless, Sr^2+^ alone exhibited a limited and gradual impact on osteogenic induction, whereas GRGDS alone demonstrates no notable positive influence on osteogenic induction. However, when strontium was combined with GRGDS, the resultant synergistic effect notably enhance osteogenesis.

### 3.4 *In vivo* osteogenesis effect of the dual-functionalization coating

**Fig. 5:**
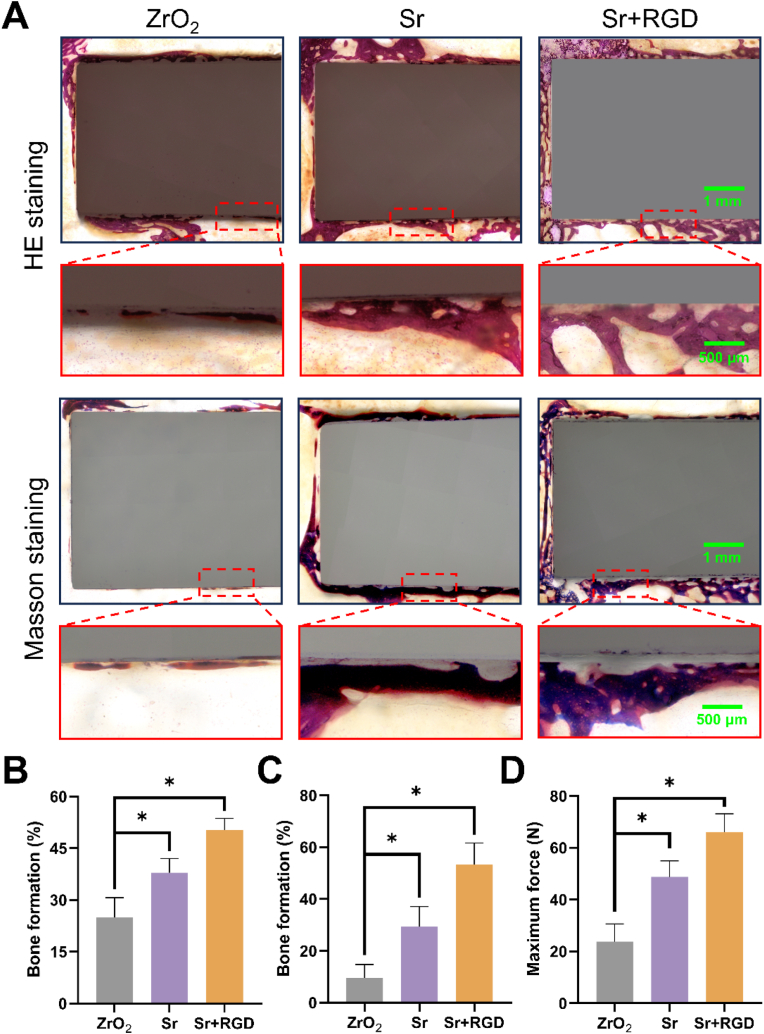
Histological analysis of (A) HE staining images and Masson staining images. (B, C) Quantitative analysis of HE and Masson staining. (D) Maximum fixation force in different samples determined by pull-out testing.

Histological analysis remains as the gold standard for evaluating implant-bone integration. Based on the result of the *in vitro*, we selected Sr and Sr + RGD group, along with the control ZrO_2_ group, to further investigate the *in vivo* bone integration of the coating using HE staining and Masson staining. The HE staining images (Fig. 5A) showed a notable augmentation in bone tissue deposition around the Sr and Sr + RGD implants, as compared to the control group. Quantitative analysis of HE staining (Fig. 5B) revealed a nearly 1.3-fold enhancement in bone formation on the Sr + RGD implant (50.3 ± 3.4%) compared to the Sr implant (37.9 ± 4.1%), and more than 2-fold enhancement compared to the ZrO_2_ implant (24.9 ± 5.8%). As illustrated in the Masson staining enhanced rate of new bone formation is evident in Sr + RGD group. Given that Masson staining exhibits a gradual color change corresponding to bone maturation (from blue to red). Depicted in Fig. 5C, the bone formation in the Sr + RGD group (53.3 ± 8.3%) was the highest among the three groups, and the Sr group (29.5 ± 7.6%) exhibited higher BIC% than the ZrO_2_ group (9.5% ± 5.2%). As shown in Fig. 5D, compared to the ZrO_2_ group (23.75 ± 6.90 N), both the Sr group and the Sr + RGD group exhibited a significant increase in maximal pull-out forces, measuring 48.79 ± 6.21 N and 65.94 ± 7.2 N, respectively. This is nearly 2.1-fold and 2.8-fold higher than that of the ZrO_2_ group. Thus, we can determine that the bone formation in Sr + RGD group appeared to have matured at a faster rate than the Sr group. In summation, Sr^2+^ has the potential to stimulate new bone formation, and the synergistic interaction with GRGDS peptide enhances the maturation of newly formed bone, which was in line with our *in vitro* results.

## 4 Conclusion

In this study, we have successfully fabricated a dual-functionalization coating on the surface of zirconia implant through a simple and effective method. The synergistic interaction between PAH and Sr enhances the aesthetic appeal and stability of the PD coating. Furthermore, PAH enriches the coating with amine groups, which offer good stability and a variety of possible chemical reactions. The results we obtained through the *in vitro* studies demonstrated that the synergistic effects of Sr^2+^ and GRGDS peptides in the coating significantly enhanced the adhesion, proliferation, and osteogenic differentiation of the MC3T3-E1 cells. Furthermore, the *in vivo* experiments also confirmed that the bio-coating on the zirconia implants could augment osseointegration to the implant. In our design, the coating not only coordinates with Sr^2+^ but also with most metal ions, such as Cu, Zn, Fe ions, etc. When combined with corresponding proteins or peptide molecules, it achieves better results or dual functionality, presenting broad prospects for application.

## CRediT authorship contribution statement

**Qihong Zhang:** Writing – original draft, Methodology, Investigation, Formal analysis, Data curation, Conceptualization. **Shuyi Wu:** Formal analysis, Data curation, Conceptualization. **Yingyue Sun:** Investigation, Formal analysis, Data curation. **Kendrick Hii Ru Yie:** Investigation, Data curation. **Jiatong Zhuang:** Investigation, Formal analysis. **Tingting Liu:** Formal analysis, Data curation. **Wen Si:** Formal analysis, Data curation. **Yinyan Zhang:** Investigation, Formal analysis. **Zheyuan Liu:** Investigation, Data curation. **Lifeng Xiong:** Data curation. **Lei Lu:** Writing – review & editing, Writing – original draft. **Peng Gao:** Data curation, Conceptualization. **Jinsong Liu:** Writing – review & editing, Writing – original draft, Validation, Supervision, Project administration, Funding acquisition.

## Declaration of competing interest

The authors declare that they have no known competing financial interests or personal relationships that could have appeared to influence the work reported in this paper.

## Data Availability

No data was used for the research described in the article.
