# Supplementary material for: Mussel byssus-inspired dual-functionalization of zirconia dental implants for improved bone integration
Source: Mater Today Bio. 2024 Feb 22;25:101007. doi: 10.1016/j.mtbio.2024.101007 (PMC11110719; doi:10.1016/j.mtbio.2024.101007)
Supplement: Multimedia component 1 [file mmc1.docx]

Supporting information

**Mussel byssus-Inspired Dual-Functionalization of** **Zirconia Dental Implants for Improved Bone Integration**

Qihong Zhang^1, †^, Shuyi Wu^1, †^, Yingyue Sun^1^, K. Hii Ru Yie^1^, Jiatong Zhuang^1^, Tingting Liu^1^, Wen Si^1^, Yinyan Zhang^1^, Zheyuan Liu^1^, Lifeng Xiong^1^, Lei Lu^1, *^, Peng Gao^1, *^, Jinsong Liu^1, *^

1*. School and Hospital of Stomatology, Wenzhou Medical University, Wenzhou, 325027, China.*

†. *These authors contributed equally.*

****. Corresponding authors:***

*Lei Lu,* [*llu2@foxmail.com*](mailto:llu2@foxmail.com)*; Peng Gao, penggaocake@foxmail.com; Jinsong Liu, jinsong0719@wmu.edu.cn.*


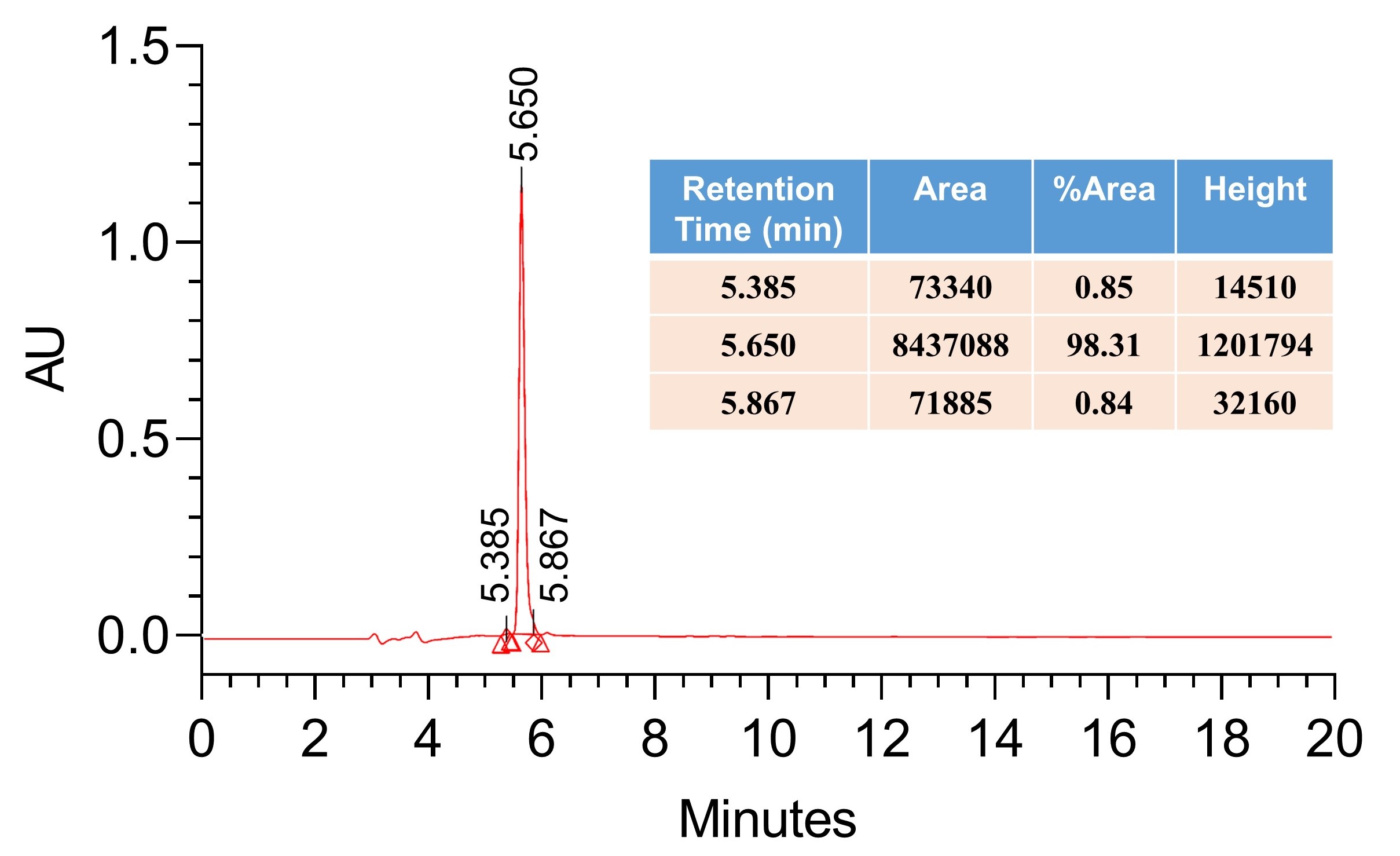


**Fig. S1** The HPLC of Mal-GRGDS (Maleimide-Glycine-Arginine-Glycine-Aspartic Acid-Serine).


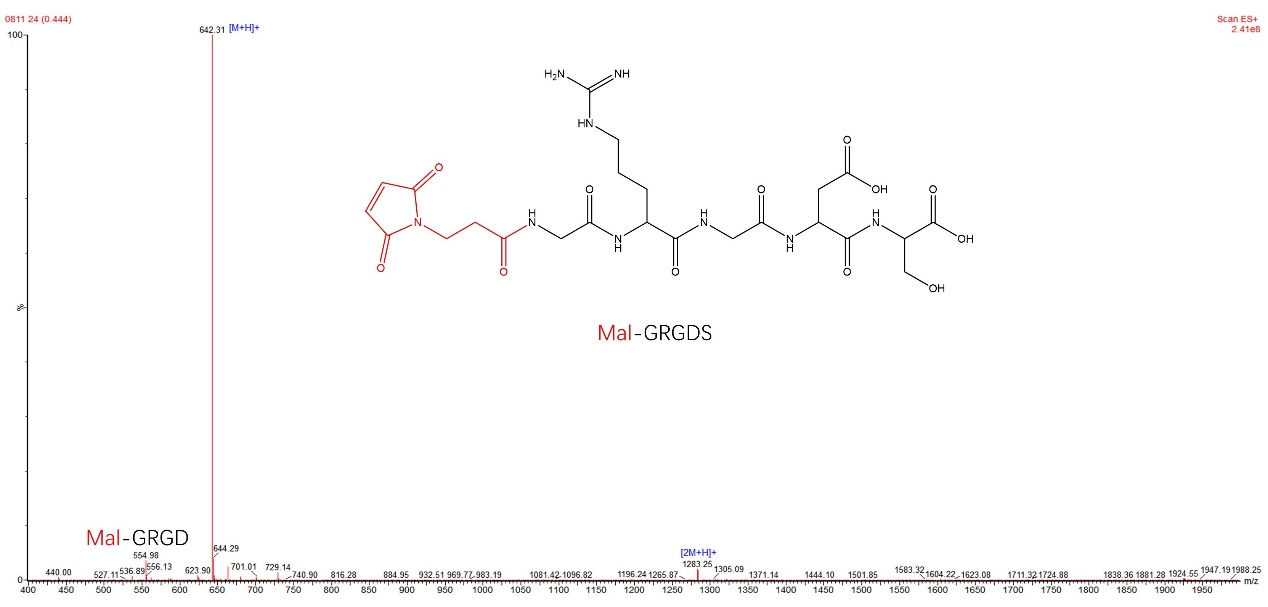


**Fig. S2** The ESI mass spectrum of Mal-GRGDS.

**
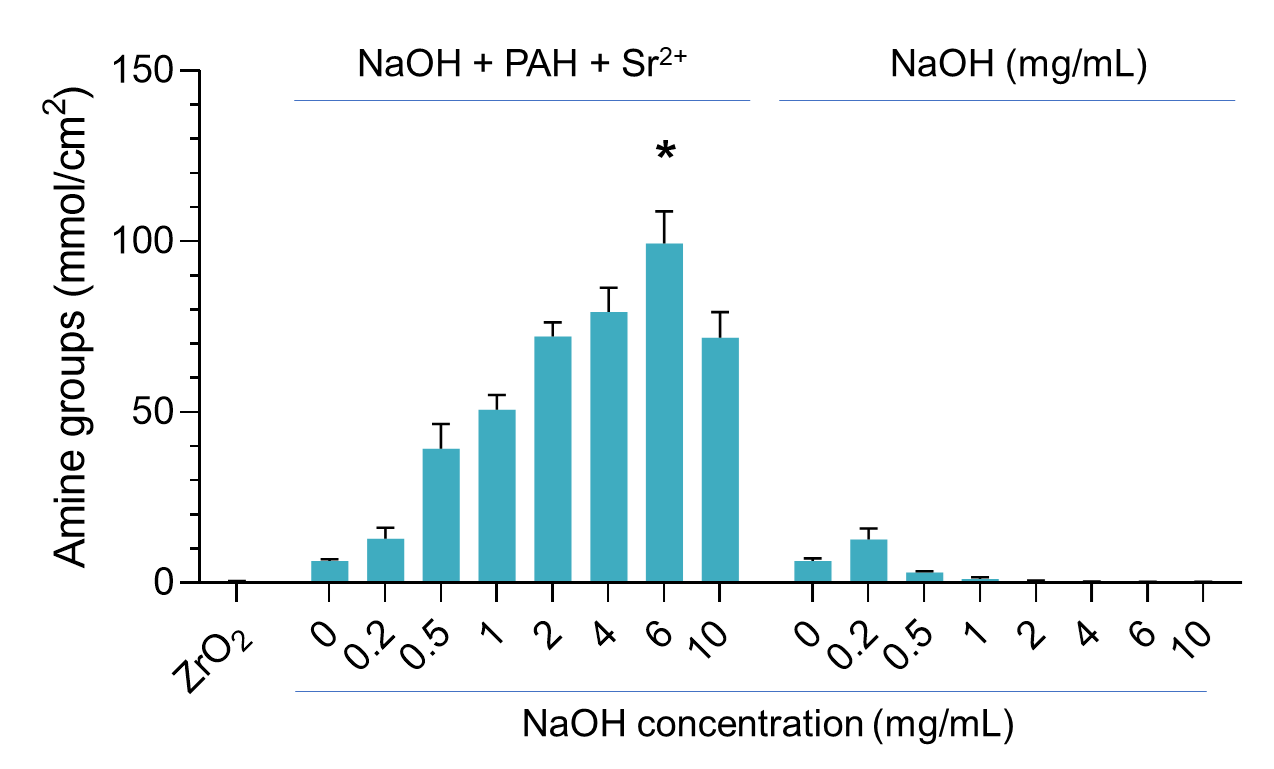
**

**Fig. S3** Relationship between NaOH concentration and the density of amine groups on the coating surface.

We explored the effect of different concentrations of NaOH on the amine groups density of the coating surface. We found that at a NaOH concentration of 6 mg/mL (measured pH of 13.2), the amine groups density on the coating surface was the highest, which is advantageous for grafting more RGD peptides.


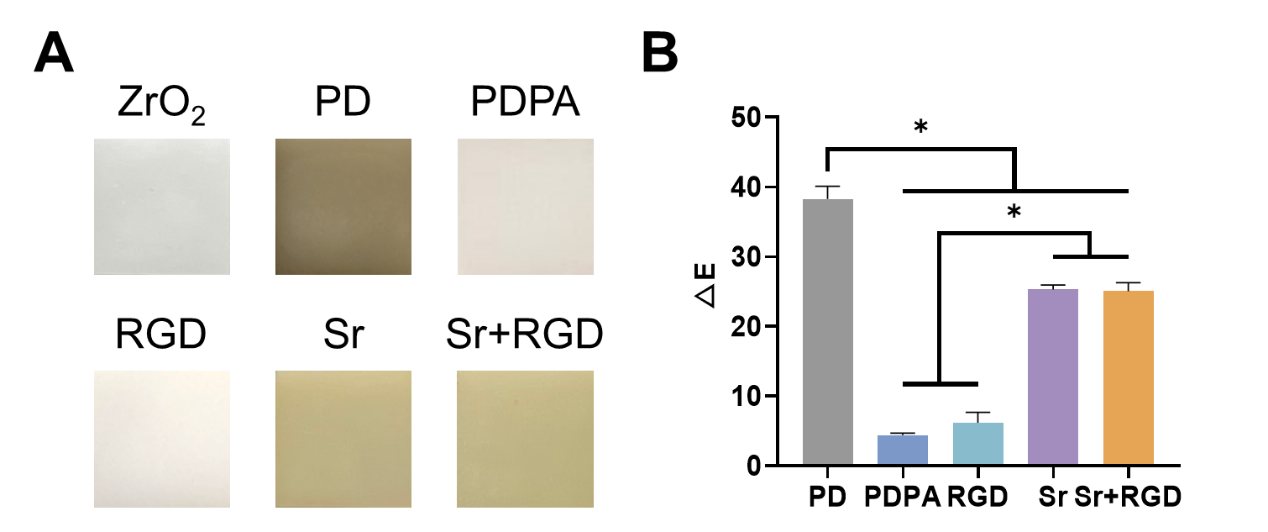


**Fig. S4** (A) The photographs of different samples, (B) quantitative analysis of color differences.


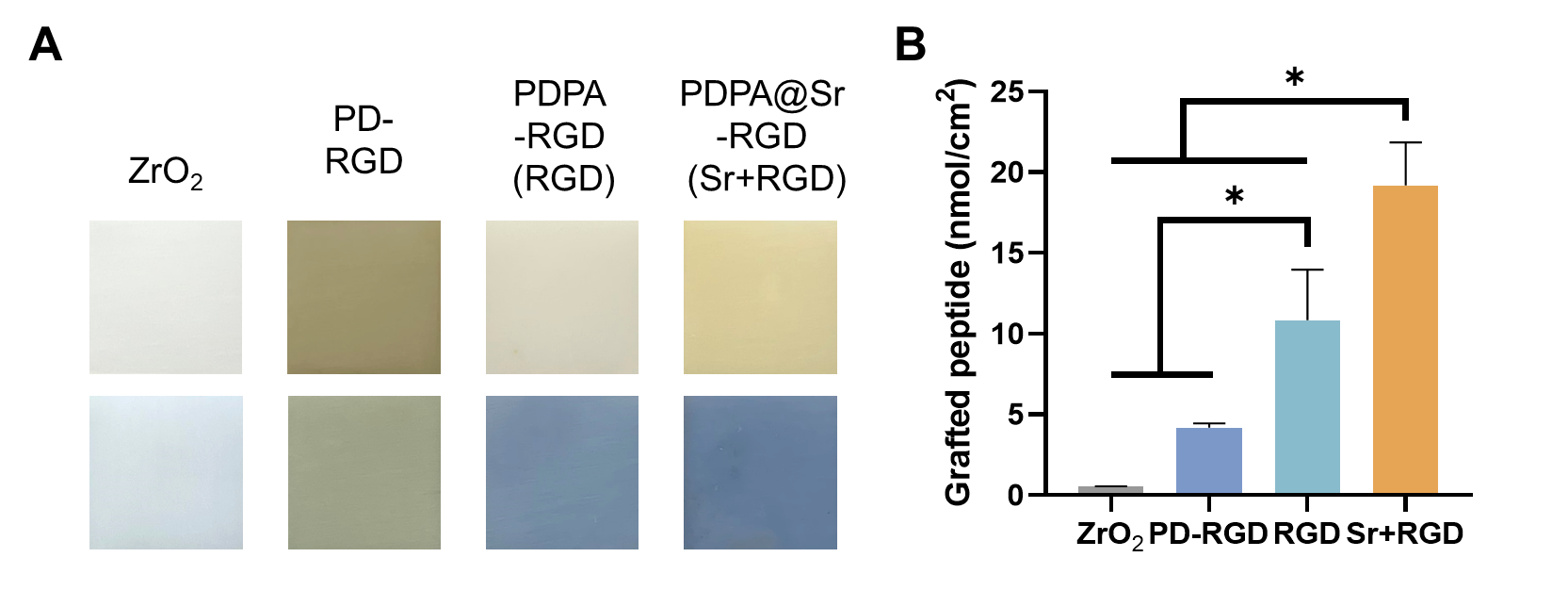


**Fig. S5** (A) Photographs of ZrO_2_, PD-RGD, PDPA-RGD, and PDPA@Sr-RGD coated surfaces before and after electrostatic adsorption of toluidine blue, along with (B) the quantitative analysis results of the grafted RGD peptide amounts.

**Table S1 Quantitative analysis of XPS.**

| **Element** | **Atomic (%)** | | | | |
| --- | --- | --- | --- | --- | --- |
|  | **ZrO_2_** | **PDPA** | **RGD** | **Sr** | **Sr+RGD** |
| Zr | 20.79 | 0.00 | 0.00 | 0.00 | 0.00 |
| C | 26.29 | 75.52 | 71.08 | 67.38 | 66.44 |
| O | 50.47 | 11.23 | 16.40 | 15.83 | 18.65 |
| N | 2.45 | 13.48 | 12.52 | 12.69 | 13.15 |
| Sr | 0.00 | 0.00 | 0.00 | 4.11 | 1.76 |
